# Implementation of an Interdisciplinary Approach to Promote Workers Global Health Status in the Oil Industry, Brazil (2006–2015)

**DOI:** 10.3390/ijerph16122148

**Published:** 2019-06-17

**Authors:** Lilian Monteiro Ferrari Viterbo, Maria Alzira Pimenta Dinis, Diogo Guedes Vidal, André Santana Costa

**Affiliations:** 1 UFP Energy, Environment and Health Research Unit (FP-ENAS), University Fernando Pessoa, 4249-004 Porto, Portugal; madinis@ufp.edu.pt (M.A.P.D.); diogovidal@ufp.edu.pt (D.G.V.); 2 Universidade Corporativa, Bahia 41745-002, Brazil; decovirtual@yahoo.com.br; 3 CNPq Research Group “Dynamics of neuro-muscle-skeletal System”, Bahiana School of Medicine and Public Health, Bahia 40290-000, Brazil

**Keywords:** worker’s health, interdisciplinary communication, chronic noncommunicable diseases (CNCDs), health promotion, oil industry

## Abstract

This study intends to analyse the behaviour of epidemiological variables of workers in an oil industry of Bahia, Brazil, before and after implementation of interdisciplinary practices in occupational health assessments between 2006 and 2015. This is a retrospective longitudinal study carried out in two time periods. Data were collected from the workers electronic medical record and time trends were analysed before (2006–2010) and after (2011–2015) the implementation of the interdisciplinary practices focusing on health promotion. The data were complementarily compared to a control group from the same industry. A statistically significant reduction for data on the number of smokers, periodontal disease and of days away from work was obtained. A significant increase in the number of physically active subjects was also observed. While not statistically significant, a reduction in the number of workers with obesity and overweight, with caries and altered glycemia, was identified. Coronary risk and high blood pressure indicators have shown aggravation. It can be concluded that an interdisciplinary health approach during the annual occupational assessments, with action directed to the population needs, can be associated with the improvement of the health indicators assessed, contributing to increased worker productivity in the oil industry.

## 1. Introduction

Chronic noncommunicable diseases (CNCDs) are one of the greatest public health problems today, responsible for 68% of the world’s deaths in 2012 [1], and for 68.3% of deaths in Brazil in 2011 [2]. The Global Action Plan for the Prevention and Control of NCDs 2013–2020 [3] from the World Health Organization (WHO) establishes some priority goals for improving the health status of the population, including reducing the prevalence of smokers, increasing prevalence of physically active persons, reducing the relative coronary risk, periodontal disease, caries, as well as controlling high blood pressure, diabetes, overweight and obesity. The Strategic Action Plan for Coping with CNCDs in Brazil from 2011 to 2022 is based on three main guidelines: (i) Surveillance, information, assessment and monitoring; (ii) health promotion; and (iii) integral care [4], ratifying the need to develop health strategies that are capable of embracing health complexity. Accordingly, the promotion of global health should be understood as a mechanism that works only with an interdisciplinary and integrated effort, aimed at an aggregating knowledge of several scientific fields that dialogue for holistic health [5]. In the context of work, CNCDs impact on the reduction of labour force participation, the number of hours worked, greater job rotation and early retirements, as well as the commitment of salaries, gains and achieved position. Estimates for Brazil suggest that the loss of labour productivity and the decrease in family income resulting from only three CNCDs, i.e., diabetes, heart disease and stroke, led to a loss in the Brazilian economy of 4.18 billion US dollars (USD) between 2006 and 2015 [6,7]. 

CNCDs are the main sources of disease burden in Brazil, and important policies for prevention and control have been implemented [7]. The Brazilian Ministry of Health [8] organized the surveillance of CNCDs aiming to respond to the scenario of growth of these pathologies in the country. This surveillance consists in a set of actions and processes allowing to know the occurrence, magnitude and distribution of the CNCDs and its main risk factors in Brazil, as well as to identify its economic, social and environmental determinants. In addition, one of the initiatives of the CNCDs surveillance aims to characterize the CNCDs time trend. These actions are essential for the planning, monitoring and evaluation of the activities of integral care and of the public policies of prevention and control of CNCDs in Brazil. The three essential components of CNCDs surveillance are: (a) Monitoring of risk factors; (b) monitoring the morbidity and mortality of CNCDs; and (c) monitoring and evaluation of health assistance and promotion actions to combat CNCDs.

The monitoring of the prevalence of CNCDs risk factors, especially those of a behavioural nature, i.e., diet, sedentary lifestyle, or chemical dependence on tobacco, alcohol and other drugs, whose scientific evidences of association with chronic diseases are proven, is one of the most important actions of surveillance [9]. The Brazilian Ministry of Health periodically invests in two important national health surveys for this purpose: National Health Survey (NHS) [10] and Surveillance of Risk Factors and Protection for Chronic Diseases by Telephone Inquiry (Vigitel) [1]. Numerous health programs and actions in Brazil converge towards the same CNCDs control objective. The Brazilian National Health Promotion Policy (PNPS) sustains that the interdisciplinary effort results in the prevention of acute and chronic diseases situations, as well as in the reduction of possible state health expenditures [11]. The Brazilian National Food and Nutrition Policy (PNAN) [12] reports that adequate food consumption and the consequent improvement of the nutritional status of citizens has a direct impact on the prevention and control of CNCDs. The Brazilian National Program to Combat Tobacco (PNCT) [13,14] is part of the solid multisectoral tobacco control policy and aims to reduce the prevalence of smokers and the consequent morbidity and mortality related to the consumption of tobacco products in Brazil [15]. The Brazilian National Oral Health Policy (PNSB) [16] aims to control oral diseases such as caries and periodontal disease, assuming that the performance of the oral health professionals should not be limited exclusively to the biological field or technical, i.e., dental, work, extending its interdisciplinary practices through education and prevention, distribution of hygiene kits, caries treatment, application of fluoride, extraction and restorations. The National Program for the Promotion of Physical Activity, “Agita Brasil” [17,18], is an initiative of the Brazilian Ministry of Health that aims to increase the knowledge of the population about the benefits of physical activity, drawing attention to its importance as a predominant factor of health protection, in order to involve citizens in the practice of such activities. In articulation with the scientific societies, i.e., Cardiology, Diabetes, Hypertension and Nephrology societies, the Brazilian Ministry of Health [19] presented the Plan for Reorganization of Attention to Hypertension and Diabetes Mellitus with the purpose of linking the patients with these diseases to the health units, guaranteeing follow-up and systematic treatment, through professionals’ training of and services reorganization. In addition to being aligned with the Global Action Plan for the Prevention and Control of NCDs 2013–2020 [3], the above-mentioned Brazilian programs have in common the character of prevention, health promotion and intersectoral actions.

The challenges for facing CNCDs in Brazil are significant and for that purpose, the articulation of actions is of fundamental importance both in the public and private sectors. Despite its rapid growth, the impact of CNCDs can be reversed through broad and cost-effective health promotion interventions to reduce the risk factors, as well as improved health care, early detection and timely treatment [20]. 

The Brazilian public policies have been effective in meeting the goals of the Strategic Action Plan for Coping with CNCDs in Brazil from 2011–2022 [4,21,22,23]. They comprise policies such as the integration and the articulation of the different sectors, organs and institutions for the construction of guidelines on CNCDs, actions in the scope of regulation of hypercaloric foods and advertisements, encouraging family farming to plant food, creation of conditioning environments for healthy living habits, agility in implementing tobacco-free environments throughout the country, and use of information as a management tool. With respect to achieving the goal of reducing the prevalence of smoking by 30% by 2022, a reduction up to 28% was already reached in 2017 and for the goal of increasing the prevalence of free-time physical activity by 10% in 2017, 23% in 2017 has already been achieved. However, the actions to curb the growth of obesity in adults need to be revised, since there was a growth up to 2017 of 25% in Brazil. 

In the current scenario of transformative health care, it is imperative that health professionals focus on care that is centred on the need of each individual in an integrated way [24]. Thus, the process of interdisciplinary work in health teams, emerging and increasingly urgent, has been supported by innovative policies, practices and care models that bring professionals and patients closer to the limits of traditional disciplinarity [25]. In this context, interprofessional education is understood as a practice of achieving interdisciplinarity as members of more than one care profession are allowed to learn together and in an interactive way, in order to improve interprofessional collaboration or the health and the well-being of patients [26]. Interprofessional collaboration has been associated with a number of positive outcomes, including improvements in patient safety and case management, optimized use of the skills of each health care team member, and provision of improved health services, identified as crucial to provision of effective and efficient health care when considering the complexity of individuals’ health needs [27]. A study conducted in Mexico by Barceló et al. [28] in 2010 identified, in some cases, improvement in glycaemic control of groups submitted to follow-up by an interdisciplinary team composed of physicians, nurses, nutritionists and psychologists, compared to groups undergoing usual care. The proportion of people with good glycaemic control (A1c < 7%) among those in the intervention group increased from 28%, before the intervention, to 39%, after the intervention. Overall, the proportion of patients achieving three or more quality improvement goals increased more than four-fold between the intervention group, from 16.6% to 69.7% (*p* < 0.01), while among the usual care group if decreased from 12.4% to 5.9% (*p* = 0.12), although not statistically significant. According to the need of interprofessional collaboration, the field of worker’s health has, since its emergence, a great potential for disciplinary integration in order to try to organize care in a more comprehensive way, translated into factors of influence on the worker’s health, difficult to achieve by the disciplines alone [29]. Occupational health assessments are essential in the examination of the health conditions of the worker and in the preservation of health by the development of the day to day work. In addition, it is the opportunity to assess the worker’s overall health, including the risk factors for CNCDs. According to the Regulatory Norm (NR) 7 [30], workers should be examined annually in the periodic assessment. However, this timeframe interval may be long for an adequate monitoring of the risks to the workers’ health. The implementation of a management policy for the risk factors of the CNCDs in a company enables the improvement of health, productivity and quality of life for all workers [6].

The main objective of this study is to describe the behaviour of epidemiological variables of a population of workers of an oil industry in Bahia, Brazil, before and after implementation of interdisciplinary health practices focusing on health promotion, carried out during annual occupational health assessments.

## 2. Materials and Methods 

### 2.1. Data Collection Procedures

This longitudinal retrospective study was developed with data collected from two periods: a first one referring to the years prior to the application of the interdisciplinary health assessment, from 2006 to 2010, and a second one, during its effective application, between 2011 and 2015. The subjects were assessed at the facilities of the occupational health service of an oil industry in Bahia, Brazil, during occupational annual assessments, in appropriate places, by health professionals with experience in the specific work area. In the period from 2006 to 2010, workers attended the oil industry’s occupational health service to perform care with professionals in the areas of medical and dental dentistry and, between 2011 and 2015 assessments with nutritionists, nurses, preventive dentistry and physical educators were included. Data with the information requested by the researchers were obtained from the oil industry, subsequently treated to standardize the variable names. A randomly generated code was created in order to guarantee the anonymity of the study participants. 

### 2.2. Study Population

The total population of workers was recruited at the occupational health service of an oil industry in Bahia, Brazil, and had, on average, the participation of 1736 subjects, starting with 1752 in 2006 and ending with 1460 in 2015. It is important to emphasize that this is a convenience sample and that, each year, the study population differed in number and composition. However, this difference between the two periods is not statistically significant, not limiting the interpretation of the results to the total population over time, which is dynamic. In all the years under study, Brazilian men, married, aged between 51 and 60 years, with administrative work regime, residing in the capital state and with high school education, prevailed in the sample. Workers included in the occupational health service and with a direct employment relationship with the oil industry studied were excluded, as well as participants with cognitive limitations or psychiatric disorders which did not allow the correct filling of electronic records, as those who were away from work.

### 2.3. Measures

The workers were annually assessed by the occupational physicians, in compliance with NR 7 [30], of the Brazilian Ministry of Health, and by the dentists, in compliance with the oil industry’s internal norm. During the study period, from 2006 to 2015, health aspects related to smoking (current smoker), activity level of the physically active (150 min of moderate physical activity during the week), relative coronary risk > 3 (difference between Assigned Risk and Average Risk > 3 – Framing Test), altered triglycerides (> 200 > 100 mg/dL), periodontal disease (Community Periodontal Index (CPI) ≥ 3), presence of caries, altered glycemia (fasting glycemia> 126 mg/dL), altered blood pressure (> 120/80 mm Hg), obesity (Body Mass index (BMI) > 30 kg/m^2^), overweight (BMI > 25 kg/m^2^) and days away from work, were evaluated. 

In the period from 2011 to 2015, interdisciplinary health promotion strategies were implemented during the annual occupational assessment, including consultations with the physical educator, nutritionist, nurse and preventive dentistry, with the objective of expanding clinical and educational opportunities for workers and improving the control of CNCDs. Collaborative health professionals explored patient priorities, providing counselling, focusing in education, and assisting with self-management goals. The physical educator promoted a complete physical assessment, using the Jackson and Pollock’s protocol of 7 folds [31], approaching aspects related to the habits of life in relation to the practice of physical activity, in order to identify what elements of the sedentary behaviour should be altered to improve the health results. Through the weekly food recall and use of educational food models, i.e., dishes, fruits, vegetables, meats, spoons, beverage cups, etc., the nutritionist focused his intervention on improving the subjects’ food profile, seeking to interfere in terms of quality improvement and adequate caloric intake. The work nurse sought to stimulate the improvement of the self-care of workers with their health, as well as to expand healthy practices in the oil industry and within family environment, addressing aspects related to active leisure, level of influence of family relationships on the individual’s health, Fagerström test for nicotine dependence [32], regular use of medications, among others. The work dentist began to act in a preventive manner, besides acting as an expert, through the inclusion of procedures such as tartarectomy, fluoride application, assisted brushing and oral examination using the intraoral chamber, enabling the worker to visualize the oral cavity and to identify areas needing greater care. In an integrated and interdisciplinary way, all health professionals guided the subjects in their needs, defining actions aiming to improve their health conditions. In addition to the practices described, an articulated set of preventive and curative health and individual actions and services were established at the levels of complexity of the worker’s health system in the oil industry, such as diet changes, creation of health promotion centres, brushstrokes, tobacco control support groups, and promotion of smoke-free environments, among several others. In summary, the test group was submitted to governmental actions, to the actions carried out by the oil industry and to the interdisciplinary strategies, which included the training of health professionals, broadening the perspective of approaching CNCDs from the perspective of health promotion and distancing from the disease by itself, aiming the self-management of chronic diseases, focusing in the worker-centred care, with the possibility of discussing cases in the interdisciplinary context and with specific referrals for each health need and discussing and assessing the effectiveness of the implemented practices.

### 2.4. Data Analysis

Data analyses were performed using the IBM^®^ SPSS^®^ Statistics 25.0 [33] (IBM, Armonk, NY, USA), considering a significance level of 0.05 for all situations of statistical inference. Descriptive statistics were calculated to characterize the continuous quantitative variables of the study, referring to risk factors and protection for chronic diseases. The oil industry workers in Brazil, excluding those in Bahia, were used as the control group for the data analysis and had, on average, the participation of 54,211 subjects, starting with 39,204 in 2006 and ending with 59,086 in 2015, a dynamic sample as already clarified.

The assessment of the impact of the health interdisciplinary practices was carried out at two levels with the application of inter and intragroup parametric tests. In the first level, the control group was compared with the test group before and after the health intervention through the Student’s *t*-test for independent samples. In the second level, the control group was compared before and after the health intervention, as well as the test group was compared before and after the health intervention through the Student’s *t*-test for paired samples. In order to complement the comparison of the means, the percentage variation of each indicator was calculated in the two groups, between the first year and the last year, respectively, 2006 and 2015. Once the first results of a health intervention project are analysed, it is important to know the trend (i.e., if indicators have been improved or, instead, were aggravated) of the health intervention in order to perceive if the changes that have been introduced lead to the initially defined goal of improving the overall health of the worker. Thus, a logarithmic prediction trend line was calculated in Excel 2016 [34] for the sample of the test group, and the results are presented for a scenario without health intervention and with health intervention.

### 2.5. Ethical Approval

In all stages of the study, the recommendations and guidelines of Resolution 466/2012 [35] of the Brazilian Ministry of Health on ethical aspects regulating research with human beings, approved by the Research Ethics Committee of the Bahia School of Medicine and Public Health and CAAE 84318218.2.0000.5544, were followed. The study included only a retrospective assessment of data available through an Institutional Database, and the analyses were performed as a part of the periodic epidemiological assessment on occupational health and safety risks. Personal data was restricted and was treated in order to guarantee the respect of privacy of the involved workers.

## 3. Results

For a reliable assessment and comparison of the results obtained with the health intervention program, it is necessary to have similar characteristics of the participants, although opting for a convenience sample. Accordingly, Table 1 shows the distribution by sex of the control and the test groups, and it was verified that despite the difference in the absolute value of the number of participants between both groups, they are similar at the percentage level, in both male and female sexes, before and after the health intervention, allowing a real comparison of the data. It should be considered that, because the participants work in the oil industry, the male population (85.0–89.3%) will tend to be larger than the female population (10.7–14.9%), a predominantly male population occupation.

The first stage of this study consisted in calculating the descriptive statistics of the indicators so that an overall analysis of the indicators would be possible, specifically to understand in which stages they were before the health intervention program. Table 2 shows that the test group had a decline in the performance of all indicators, except for smokers (−0.6%), when compared to the control group. 

As mentioned previously, the evaluation of the results of the health intervention in the test group was performed at two levels, inter and intragroup (Table 3). The three most worrying indicators in the test group are the periodontal disease (10.2 ± 0.01%), high blood pressure (19.8 ± 3.19%) and high glycaemia (22.7 ± 5.31%), which are very high, when compared to the control group, respectively 5.3 ± 0.00, 13.1 ± 0.69 and 17.3 ± 0.77, between 2006 and 2015. These indicators require immediate intervention in global health, since when combined they can lead to the development of several serious chronic diseases.

The results in Table 4 show that, in the case of the test group smokers and physically active, the indicators follow a similar evolution to the one observed in the Brazilian data (i.e., the smokers decrease and the physically active increase in both cases). On the other hand, a reduction in the obesity and overweight (−0.07%), and also, in the high glycaemia (−0.35%) indicators were identified in the test group. Relating to the same indicators, Brazilian data reveal an increase, respectively, of 1.28% and 0.23%. An increase in the high blood pressure indicator was identified both in test group and in the Brazilian data.

**Figure 1:**
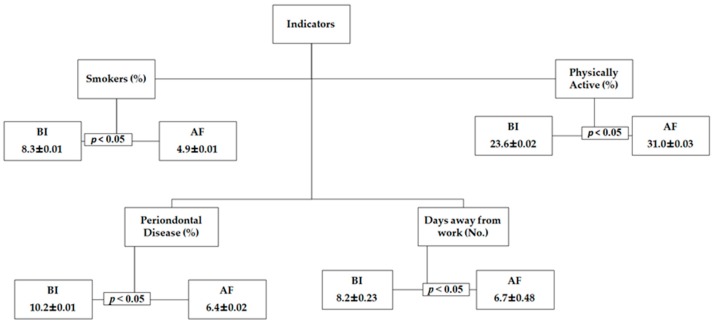
Group indicators corresponding to significant statistical differences after health intervention. Note: (BI) Before Intervention; (AF) After Intervention. Significant differences are presented in bold (*p* < 0.05).

In order to synthesize the results presented in the Table 3 and Table 4, Figure 1 presents the indicators of the test group, corresponding to a statistically significant difference after the health intervention.

Table 5 presents a comparative analysis of the prevalence of smoking, physically active and obesity among Brazilian workers, the control group and the test group, based on the goals of the Strategic Action Plan for Coping with CNCDs in Brazil from 2011 to 2022, planned by the Brazilian Ministry of Health [4].

The test group stands out in the evolution of the three analysed goals, showing an increase in the prevalence of physical activity in free time by 50% between 2010 and 2017, a percentage well above the proposed target defined in the Strategic Action Plan for Coping with CNCDs in Brazil from 2011 to 2022 [4] of 10% up to 2022. The same positive result can be observed in the control of obesity growth, where both Brazil and the control group show a significant percentage of growth and the test group reaches the goal of stabilizing this health indicator. Regarding the goal of reducing smoking prevalence by 30%, the overall goal in Brazil (28%) and a significant advance for the test group were observed, reaching a reduction of 56%, also above the control group (53%).

Aiming to summarize the evolution trend of all health indicators, Table 6 presents the obtained results of the indicators in the test group, after health intervention.

To each indicator a negative or positive polarity was attributed according to the impact on the worker’s health, i.e., smoking has a negative polarity (–) due to its negative impacts on health and physical activity has a positive polarity (+) due to its positive impacts on health. An indicator trend was added to better understand the evolution direction, namely if increasing or decreasing, i.e., coronary risk had increased (↑) and caries had decreased (↓). Finally, an assessment was carried out aiming to identify which indicators had suffered improvement or aggravation since the beginning of the intervention, i.e., periodontal disease has a negative polarity (–) and its trend is to decrease (↓), which represents an improvement (Improvement). On the other hand, coronary risk has a negative polarity (–) and its trend is to increase (↑), representing an aggravation (Aggravation).

Table 6 shows that, in general, interdisciplinary health intervention in the global worker’s health reveals positive results in seven of the nine indicators assessed, namely: decrease of the percentage of smokers, increase of the physically active, decrease of the periodontal disease rate, decrease in the percentage of obese people and overweight, decrease in the number of caries, decrease in the number of workers with high glycaemia and a decrease in the number of days away from work. The remaining indicators where no improvements were observed, namely coronary risk and high blood pressure, should be considered as priorities for subsequent health interventions. 

**Figure 2:**
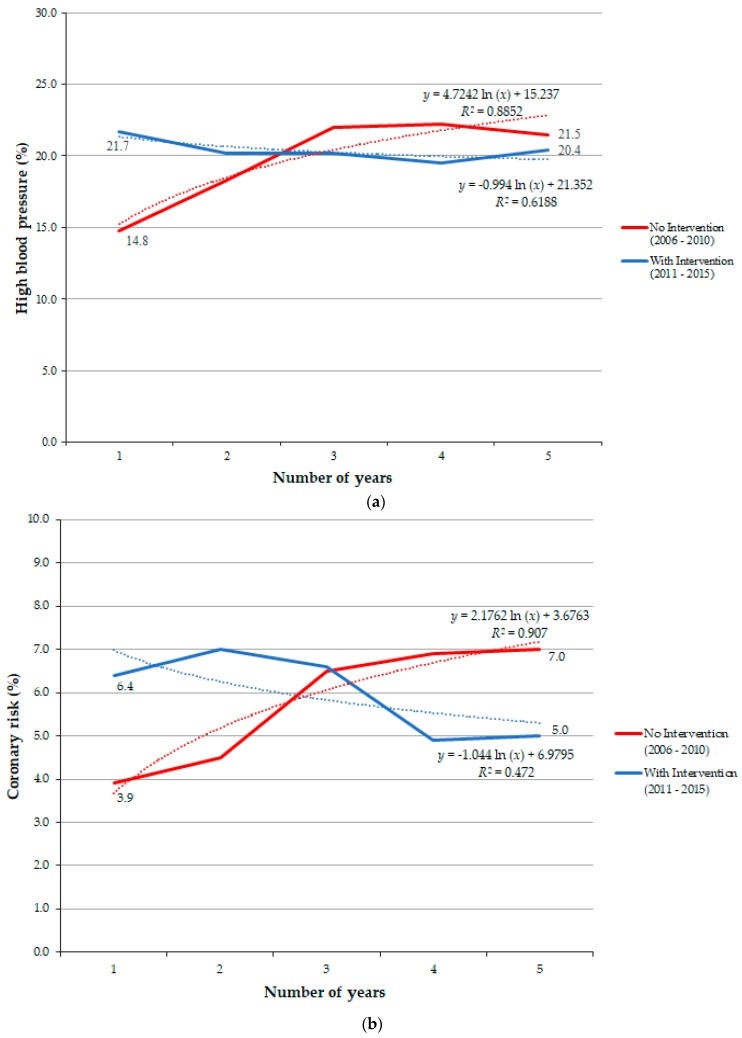
Prediction trend lines corresponding to indicators in which no improvements were observed (**a**) evolution of the high blood pressure indicator, (**b**) evolution of the coronary risk indicator.

Intending to present the results of the indicators in which no improvement was observed, a logarithmic prediction trend line—to a five year period—was calculated in two different scenarios for each indicator, with and without health intervention, Figure 2.

The starting values in Figure 2 are different in the two prediction lines due to the fact they are based in the real baseline values of the two different moments: Before intervention and after intervention. The results show that although no significant differences were found in the two types of indicators, the scenario would be aggravated if interdisciplinary health intervention was not implemented.

## 4. Discussion

According to the Brazilian health targets to control CNCDs [4], the difference between the results in the different levels of interventions are accomplished: at a public level, through the public policies of prevention and control of CNCDs in Brazil, at a private level with broad actions of health promotion being carried out, i.e., corresponding to the control group, and at a private level with interdisciplinary interventions directed to the target population, i.e., corresponding to the test group. The positive results obtained in the test group must be emphasised, reinforcing the understanding that the interdisciplinary health practices have positively affected the global health of the studied population.

Table 2 shows that the test group presented a higher prevalence for diseases in the initial study period (2006–2010), except for the indicators of smoking and obesity and overweight, which were slightly aggravated in the control group. After the implementation of the interdisciplinary interventions (2011–2015), an improvement of the profile of the test group for all the health indicators, with exception of the coronary risk and high blood pressure indicators, is observed, which may be justified by sociodemographic aspects such as sex, age and race, as reported by Khera et al. [36], as well as cultural, whose context is very peculiar in Bahia, Brazil. The results of Table 3 reinforce the results present in Table 2 and report the improvements achieved by the test group in relation to the control group for all health indicators except coronary risk and high blood pressure. Regarding smoking, there was an equivalent reduction between both the test and the control groups, ranging from −0.61 to 0.60, without significant differences, this is explained by the fact that the Program to Combat Tobacco is interdisciplinary and applied in a similar way to both test and control groups. Table 4 and Table 5 present the advances obtained in the test group in relation to the Brazilian population for all comparable indicators, except for blood pressure. One of the main objectives of the interdisciplinary intervention is not be to seek that the test group presents better results than the control group, but that the results of the test group approach those of the control group, thus representing a global improvement in the overall health profile of the workers test group.

The sedentary lifestyle causes about three million or 8% of all CNCDs due annual deaths in the world [6]. The benefits of an active lifestyle and the education of workers are essential for the promotion of physical activity and overcoming the barriers commonly reported for this practice, such as the lack of time and access to adequate spaces for the practice of exercise [37,38]. In the period under analysis (2006–2015), a growth of the physically active workers in the mean annual variation of 0.51% (Table 3) per year was observed in the test group, being above the growth variation of the physically active in the control group (0.40%), in accordance with the Brazilian population trend, which was 0.24% per year. These results show that it is possible to change behaviours that meet healthier lifestyles, able to be achieved through structured planning by an interdisciplinary team and centred in the individual. According to Lin (2014), the work context can and should function as an institution that promotes the overall health of the worker.

The obesity epidemic that affects the world, with the consequent increase in the prevalence of diabetes and hypertension, threatens the further reduction of CNCDs [7]. Obesity and overweight are associated with an increased risk of morbidity and mortality due to hypertension, dyslipidaemia, diabetes mellitus and cardiovascular diseases [39]. In this study, the test group presented a higher percentage of workers with obesity and overweight, when compared to the control group, and a more intense percentage variation in the level of overweight reduction after implementation of the interdisciplinary health interventions in the oil industry, whereas there was a growth in the variation of 1.28%in the Brazilian population.

According to Brazilian Ministry of Health [18], cardiovascular diseases are the main cause of morbidity and mortality in the Brazilian population. There is no single cause for these diseases, but several risk factors, which increase the probability of their occurrence. High blood pressure and diabetes mellitus represent two of the main risk factors, contributing decisively to the aggravation of this scenario at the national level [40]. In this study, it was observed that the test group presented a higher percentage of workers with high blood pressure compared to the control group (Table 3), as well as an increase in the analysed period of 2006 to 2015, reinforcing the need for more specific health intervention actions for this specific population. With regard to altered glycaemia, a higher prevalence was observed in the test group, in addition to the more marked variation in the reduction of the percentage of workers in this group, in the order of −0.35%, whereas the control group varied in −0.23% and the Brazilian population in 0.23%. Although the results of the study for the high cardiovascular risk were not statistically significant, there was a stagnation of the data variation, which shows a control of cardiovascular risk behaviour, emphasizing the efficiency of the health practices implemented, namely at an interdisciplinary level. The study shows that cardiovascular risk and high blood pressure present better results after the interdisciplinary health intervention, indicating a tendency for improvement in both cases.

As a risk factor for the development of a number of chronic diseases related to cancer, lung diseases and cardiovascular diseases, smoking continues to lead the causes of avoidable global deaths in the world [41]. Brazil stands out in the implementation of tobacco control measures in the world, along with countries like Australia, Canada, Panama, Turkey and Uruguay [42] and the success of the Brazilian tobacco control policy between 1986 and 2016 is evidenced by the expressive reduction in the prevalence of smokers over those years [43]. The PNCT follows a model of interdisciplinary action involving physicians, nurses, psychologists, dentists, among others, in which educational, communication and health care actions [15], along with support for adoption or compliance with legislative and economic measures, are potentiated to prevent the initiation of smoking, especially among adolescents and young people, to promote smoking cessation and to protect the population from exposure to environmental tobacco smoke, also reducing the individual, social and environmental damage of tobacco products [9,44]. The PNCT has excellent results and in the study period of 2006 to 2015, the reduction of smokers in the mean annual variation of −0.60% per year in the test group is observed, above the variation of decrease of the Brazilian population, which was −0.33% per year [45], ratifying the importance of the targeted actions developed in the studied oil industry, and recommended in this study. 

According to the Pan American Health Organization (PAHO) (2016) [46], in the last decade, scientific evidence of the connection between oral health and systemic disease has continued to grow, making oral health an important component of disease prevention in public health. Behavioural risk factors related to oral diseases are common to other major CNCDs, including an unhealthy diet rich in free sugars, smoking, and harmful alcohol consumption [47,48]. Periodontal disease, an infectious pathology with a multifactorial cause, affects the periodontal tissues and is related to diabetes [49], cardiovascular diseases [50] and stress [51]. In 2010, the prevalence of Brazilians with periodontal disease was 22.7%, in the age group of 35–74 years old, which is high when compared to the sample of this study, which presents a reduction of 4% (8% in 2006 for 4.2% in 2015), after the implementation of interdisciplinary health practices. The test group presents a higher percentage of workers with caries (0.9 ± 0.74%) compared to the control group (2.3 ± 2.16%), however it presents an average annual variation of −0.50% years, being more intense in the reduction of the disease in the studied period of 2006 to 2015, while there was an increase in the number of workers with caries in the control group in the range of 0.06% (Table 3). 

The increase in the prevalence of cases of CNCDs in the oil industry can result in a reduction in productivity, absenteeism, disability, early retirement and increased expenses on the health system. The management of risk factors for CNCDs is essential to guarantee workers’ overall health [52]. It was observed in this study that the test group presented a greater number of days away from work of workers when compared to the control group before the health intervention period of 2006 to 2010. After the health intervention, the test group was the only group that was able to reverse this trend, with a reduction of the number of days away from work in the range of −0.27% per year, oppositely to the control group, which increased by 0.10% per year (Table 3). 

It is clear that a possible scenario without health intervention programs would aggravate performance in all studied health indicators, meaning that interdisciplinary health interventions have and will have very positive and relevant impacts in the short, medium and long term of worker’s health, and it is crucial to continue to invest in actions to assist the main objective, i.e., improving the well-being and the overall health of the worker.

This study is of great epidemiological importance since it deals with the database on workers of an important Brazilian oil industry, thus reflecting two scenes in the studied context universe. First, the national scene of skilled oil workers in the period of 2006 to 2015, typically reported by strata of national surveys, such as Vigitel [1]. Then, it also reflects the level of intervention of health and work technicians, including all professions or disciplines involved, reporting what changes were introduced in relation to the external universe so that the target public, i.e., the test group, is considered under the intervention of the team, besides the intervention that the population undergo in the same period.

It is important to deepen the research of the variables that did not correspond to significant statistical changes, aiming at a better understanding of the scenario such as race, sex, environmental, labour and cultural determinants under analysis. Another relevant aspect is the analysis of the technical and economic viability of the implementation of interdisciplinary health practices, essential in the socioeconomic context of Brazil. Studies of Mendes [53], Bielemann [54] and Djalalov [55] have demonstrated the importance of investing in health promotion.

## 5. Conclusions

During the period 2006 to 2015 under study there was a strong investment in public policies to control the CNCDs in Brazil. Changes were adopted by different domains of society either public or private institutions. In accordance to this, the oil industry incorporated company wide initiatives and allowed healthier life practices to be stimulated in the labour context, generating positive and improved health outcomes, when compared to the ones corresponding to the Brazilian population. The oil Worker Health Service studied in Bahia, Brazil, carried out specific health strategies, integrating interdisciplinary health interventions centred on the worker, which allow a personalized monitoring of individual needs of workers, something that Brazilian public policies are not able to accomplish. Reported positive results presented in this study are able to sustain the success of such initiatives. It is important to note that the main objective of this interdisciplinary health approach was achieved, namely the improvement of the test group health indicators, with exception of two interconnected indicators: coronary risk and high blood pressure, whose negative results are explained considering the workers sociodemographic profile. The logarithmic prediction trend lines indicate that, in the future, this type of interdisciplinary approach centred in the needs of workers will result in potential health gains, namely in the reduction of workers with coronary risk and high blood pressure, two of the main causes of death in the world.

The results in this study show that the interdisciplinary and integrated approach during occupational health assessments, aiming to satisfy the specific needs of oil workers, are associated with the improvement of the global health indicators, thus representing a positive outcome to this specific industry.

## Figures and Tables

**Table 1:** Sample characterization by sex before and after the implementation of the health intervention program.

Stage	Year	Control Group			Test Group		
		Total (*N*)	Male (%)	Female (%)	Total (*N*)	Male (%)	Female (%)
Before Intervention (BI)	2006	39,204	87.7	12.3	1574	89.3	10.7
	2007	47,152	86.5	13.5	1727	88.9	11.1
	2008	49,140	86.2	13.8	1724	88.9	11.1
	2009	54,269	85.7	14.3	1792	88.9	11.1
	2010	54,537	85.8	14.2	1778	88.6	11.4
After Intervention (AF)	2011	56,645	85.7	14.3	1810	88.5	11.5
	2012	59,836	85.4	14.7	1831	88.7	11.3
	2013	61,492	85.1	14.9	1805	88.4	11.6
	2014	60,753	85.1	14.9	1784	88.5	11.5
	2015	59,086	85.0	14.9	1591	88.2	11.8

**Table 2:** Indicators’ descriptive statistics before and after the implementation of the health intervention program.

Indicator	Stage	Control Group		Test Group	
		Mean ± Std.	Min–Max	Mean ± Std.	Min–Max
Smokers (%)	BI	8.9 ± 0.01	7.4–10.9	8.3 ± 0.01	7.1–9.9
	AF	5.4 ± 0.01	4.3–6.8	4.9 ± 0.01	3.8–6.6
Physically Active (%)	BI	24.4 ± 0.03	21.5–27.6	23.6 ± 0.02	21.4–25.4
	AF	29.1 ± 0.01	28.0–30.2	31.0 ± 0.03	26.6–33.8
Coronary Risk (%)	BI	4.2 ± 0.00	4.1–4.3	5.8 ± 0.01	3.9–7.0
	AF	3.8 ± 0.01	3.2–4.3	6.0 ± 0.00	4.9–7.0
Periodontal Disease (%)	BI	5.3 ± 0.00	4.8–5.6	10.2 ± 0.01	8.1–11.5
	AF	3.6 ± 0.01	2.8–4.6	6.4 ± 0.02	4.0–9.1
Obesity and Overweight (%)	BI	69.2 ± 0.04	65.5–75.0	71.5 ± 0.02	69.5–74.7
	AF	65.2 ± 0.03	61.2–68.1	69.8 ± 0.04	63.6–72.9
Caries (%)	BI	0.9 ± 0.74	0.02–1.73	2.3 ± 2.16	0.02–4.89
	AF	1.6 ± 0.30	1.27–2.02	3.3 ± 1.41	1.79–5.35
High Blood Pressure (%)	BI	13.1 ± 0.69	12.3–13.9	19.8 ± 3.19	14.8–22.2
	AF	12.4 ± 1.07	11.2–13.7	20.4 ± 0.80	19.5–21.7
High Glycaemia (%)	BI	17.3 ± 0.77	16.1–18.0	22.7 ± 5.31	17.3–29.7
	AF	16.0 ± 1.74	13.5–17.5	22.1 ± 3.62	17.2–25.9
Days Away from Work (No)	BI	6.4 ± 0.48	5.85–6.83	8.2 ± 0.23	7.95–8.55
	AF	6.7 ± 0.16	6.51–6.83	6.7 ± 0.48	6.21–7.39

Note: (BI) Before Intervention; (AF) After Intervention.

**Table 3:** Mean comparison within and among control and test groups.

Indicators	Stage	Control Group (*Mean*)^a^	*p*^1–2^**	Test Group (*Mean*)^b^	*p*^3–4^**	Percent Variation (%)	
						Control Group	Test Group
Smokers	BI	**8.9^1^**	**0.00**	**8.3^3^**	**0.00**	−0.61	−0.60
	AF	5.4^2^		4.9^4^			
	BI	*p*^a–b^ *		0.22			
	AF			0.26			
Physically Active	BI	24.4^1^	**0.00**	23.6^3^	**0.00**	0.40	0.51
	AF	29.1^2^		31.0^4^			
	BI	*p*^a–b^*		0.30			
	AF			0.10			
Coronary Risk	BI	4.2^1^	0.08	5.8^3^	0.45	−0.27	0.30
	AF	3.8^2^		6.0^4^			
	BI	*p*^a–b^*		**0.02**			
	AF			**0.00**			
Periodontal Disease	BI	5.3^1^	**0.01**	10.2^3^	**0.02**	−0.42	−0.62
	AF	3.6^2^		6.4^4^			
	BI	*p*^a–b^*		**0.00**			
	AF			**0.01**			
Obesity and Overweight	BI	69.2^1^	**0.02**	71.5^3^	0.20	−0.17	−0.07
	AF	65.2^2^		69.8^4^			
	BI	*p*^a–b^*		0.13			
	AF			**0.03**			
Caries	BI	0.9^1^	**0.04**	2.3^3^	0.19	0.06	−0.50
	AF	1.6^2^		3.3^3^			
	BI	*p*^a–b^*		0.10			
	AF			**0.01**			
High Blood Pressure	BI	13.1^1^	0.22	19.8^3^	0.37	−0.09	0.38
	AF	12.4^2^		20.4^3^			
	BI	*p*^a–b^*		**0.00**			
	AF			**0.00**			
High Glycaemia	BI	17.3^1^	0.09	22.7^3^	0.38	−0.23	−0.35
	AF	16.0^2^		22.1^4^			
	BI	*p*^a–b^*		**0.03**			
	AF			**0.00**			
Days Away from Work	BI	6.4^1^	0.18	8.2^3^	**0.00**	0.10	−0.27
	AF	6.7^2^		6.7^4^			
	BI	*p*^a–b^*		**0.00**			
	AF			0.45			

Note: (BI) Before Intervention; (AF) After Intervention. Significant differences are presented in bold (*p*<0.05). ^a^ control group and ^b^ test group intergroup means comparison; ^1^AI and ^2^ BI intragroup comparison (control group); ^3^AI and ^4^BI intragroup comparison (test group); *independent sample *t*-test; ** paired sample *t*-test.

**Table 4:** Percent variation comparison between the test group and the Brazilian workers (2006–2015).

Indicator (%)	Test Group	Brazil
Smokers	−0.60	−0.33
Physically Active	0.51	0.24
Obesity and Overweight	−0.07	1.28
High Blood Pressure	0.38	0.22
High Glycaemia	−0.35	0.23

**Table 5:** Comparative analysis based on the goals of the Strategic Action Plan for Coping with CNCDs in Brazil from 2011 to 2022. (Results with the best performance are in bold).

Brazil CNCDsPlan Goals (2011–2022)	Baseline Value (2010)	Latest Result (2017)	Evolution	Group
Smoking prevalence reduction by 30 %	14.1	10.1	−28	Brazil
	7.4	3.5	−53	Control group
	7.1	3.1	**−56**	Test group
Increase in the prevalence of physical activity practice in free time by 10%	30.1	37	23	Brazil
	27.6	30.8	11	Control group
	25.4	38.1	**50**	Test group
Control of the increase in obesity in adults %	15.1	18.9	25	Brazil
	22.03	24.04	9	Control group
	24.33	24.61	**1**	Test group

**Table 6:** Test group indicators’ assessment after health intervention (2006–2015).

Indicator	Polarity	Trend	Assessment
Smokers	−	↓	Improvement
Physically Active	+	↑	Improvement
Coronary Risk	−	↑	Aggravation
Periodontal Disease	−	↓	Improvement
Obesity and Overweight	−	↓	Improvement
Caries	−	↓	Improvement
High Blood Pressure	−	↑	Aggravation
High Glycaemia	−	↓	Improvement
Days Away from Work	−	↓	Improvement

Note: ↑ – increased; ↓ – decreased.
